# Factors Associated With Household Transmission of SARS-CoV-2: An Updated Systematic Review and Meta-analysis

**DOI:** 10.1001/jamanetworkopen.2021.22240

**Published:** 2021-08-27

**Authors:** Zachary J. Madewell, Yang Yang, Ira M. Longini, M. Elizabeth Halloran, Natalie E. Dean

**Affiliations:** 1 Department of Biostatistics, University of Florida, Gainesville; 2 Fred Hutchinson Cancer Research Center, Seattle, Washington; 3 Department of Biostatistics, University of Washington, Seattle

## Abstract

**Importance:**

A previous systematic review and meta-analysis of household transmission of SARS-CoV-2 that summarized 54 published studies through October 19, 2020, found an overall secondary attack rate (SAR) of 16.6% (95% CI, 14.0%-19.3%). However, the understanding of household secondary attack rates for SARS-CoV-2 is still evolving, and updated analysis is needed.

**Objective:**

To use newly published data to further the understanding of SARS-CoV-2 transmission in the household.

**Data Sources:**

PubMed and reference lists of eligible articles were used to search for records published between October 20, 2020, and June 17, 2021. No restrictions on language, study design, time, or place of publication were applied. Studies published as preprints were included.

**Study Selection:**

Articles with original data that reported at least 2 of the following factors were included: number of household contacts with infection, total number of household contacts, and secondary attack rates among household contacts. Studies that reported household infection prevalence (which includes index cases), that tested contacts using antibody tests only, and that included populations overlapping with another included study were excluded. Search terms were *SARS-CoV-2* or *COVID-19* with *secondary attack rate*, *household*, *close contacts*, *contact transmission*, *contact attack rate*, or *family transmission*.

**Data Extraction and Synthesis:**

Meta-analyses were performed using generalized linear mixed models to obtain SAR estimates and 95% CIs. The Preferred Reporting Items for Systematic Reviews and Meta-analyses (PRISMA) reporting guideline was followed.

**Main Outcomes and Measures:**

Overall household SAR for SARS-CoV-2, SAR by covariates (contact age, sex, ethnicity, comorbidities, and relationship; index case age, sex, symptom status, presence of fever, and presence of cough; number of contacts; study location; and variant), and SAR by index case identification period.

**Results:**

A total of 2722 records (2710 records from database searches and 12 records from the reference lists of eligible articles) published between October 20, 2020, and June 17, 2021, were identified. Of those, 93 full-text articles reporting household transmission of SARS-CoV-2 were assessed for eligibility, and 37 studies were included. These 37 new studies were combined with 50 of the 54 studies (published through October 19, 2020) from our previous review (4 studies from Wuhan, China, were excluded because their study populations overlapped with another recent study), resulting in a total of 87 studies representing 1 249 163 household contacts from 30 countries. The estimated household SAR for all 87 studies was 18.9% (95% CI, 16.2%-22.0%). Compared with studies from January to February 2020, the SAR for studies from July 2020 to March 2021 was higher (13.4% [95% CI, 10.7%-16.7%] vs 31.1% [95% CI, 22.6%-41.1%], respectively). Results from subgroup analyses were similar to those reported in a previous systematic review and meta-analysis; however, the SAR was higher to contacts with comorbidities (3 studies; 50.0% [95% CI, 41.4%-58.6%]) compared with previous findings, and the estimated household SAR for the B.1.1.7 (α) variant was 24.5% (3 studies; 95% CI, 10.9%-46.2%).

**Conclusions and Relevance:**

The findings of this study suggest that the household remains an important site of SARS-CoV-2 transmission, and recent studies have higher household SAR estimates compared with the earliest reports. More transmissible variants and vaccines may be associated with further changes.

## Key Points

**Question:**

Are early estimates of household transmission of SARS-CoV-2 indicative of current household transmission?

**Findings:**

In this updated systematic review and meta-analysis of 87 studies representing 1 249 163 household contacts from 30 countries, the estimated household secondary attack rate was 19%. An increase in household transmission was observed over time, perhaps owing to improved diagnostic procedures and tools, longer follow-up, more contagious variants, and different study locations.

**Meaning:**

These findings suggest that the household remains an important site of SARS-CoV-2 transmission, and recent studies have generated higher household secondary attack rate estimates compared with the earliest reports; more transmissible variants and vaccines may be associated with additional changes in the future.

## Teaser

This systematic review and meta-analysis combines data from a previous meta-analysis of SARS-CoV-2 household transmission through October 19, 2020, with new data from studies published between October 20, 2020, and June 17, 2021, to provide updated estimates of household secondary attack rates.

## Introduction

Understanding of the household secondary attack rate for SARS-CoV-2 is still evolving. We previously published a systematic review and meta-analysis of household transmission of SARS-CoV-2 that summarized 54 published studies representing 77 758 household contacts through October 19, 2020, finding an overall secondary attack rate (SAR) of 16.6% (95% CI, 14.0%-19.3%).^1^ Household SARs were higher to adult contacts than to child contacts, to spouses than to other contacts, from symptomatic index cases than from asymptomatic index cases, and in households with 1 contact than in households with 3 or more contacts. The SARs were higher to household contacts than to other close contacts. Household SARs were also higher for SARS-CoV-2 than for SARS-CoV and Middle East respiratory syndrome coronavirus. This living systematic review and meta-analysis updated those findings through June 17, 2021, and used newly published data to further our understanding of the household’s role in SARS-CoV-2 transmission.^2^

## Methods

This study followed the Preferred Reporting Items for Systematic Reviews and Meta-analyses (PRISMA) reporting guideline using the same definitions, search strategy, eligibility criteria, and data extraction methods used in our original study.^1^ We searched PubMed and reference lists of eligible articles for studies published between October 20, 2020, and June 17, 2021, with no restrictions on language, study design, time, or place of publication. Studies published as preprints were included. Search terms were *SARS-CoV-2* or *COVID-19* with *secondary attack rate*, *household*, *close contacts*, *contact transmission*, *contact attack rate*, or *family transmission*.

Articles with original data that reported at least 2 of the following factors were included: number of household contacts with infection, total number of household contacts, and secondary attack rates among household contacts. Studies that reported household infection prevalence (including index cases), that tested contacts using antibody tests only, and that included populations that overlapped with another included study were excluded.

In addition to the covariates examined previously, we also examined SAR by contact ethnicity (restricted to studies in the US), contact comorbidity, index case fever, index case cough, and variant (if reported in ≥3 studies). Primary outcomes were overall household SAR for SARS-CoV-2, SAR by covariates (contact age, sex, ethnicity, comorbidities, and relationship; index case age, sex, symptom status, presence of fever, and presence of cough; number of contacts; study location; and variant), and SAR by index case identification period. We categorized contact and index case age as adults (aged ≥18 years) and children (aged <18 years). For studies that reported SARs by age using 10-year increments (eg, 10-19 years), we included those aged 18 and 19 years in the child category. For the symptom status of the index case covariate, we included studies that disaggregated SARs for at least 2 of the following: symptomatic, presymptomatic, and asymptomatic individuals. We also conducted a sensitivity analysis restricted to studies with a more uniform design, which excluded studies with only asymptomatic or pediatric index cases, studies that tested only symptomatic or asymptomatic contacts, studies with long follow-up periods (≥21 days), and studies published as preprints.

In addition, to examine temporal patterns, we assessed household SARs by index case identification period (January-February 2020, March-April 2020, May-June 2020, and July 2020-March 2021). If the study period spanned multiple months, we used the midpoint. For example, when the index case identification period for all households was December 2019 to April 2020, the midpoint was February 2020, and the study was categorized as January to February 2020.

### Statistical Analysis

Statistical analyses were similar to those previously described.^1^ However, this analysis used generalized linear mixed models to obtain SAR estimates and 95% CIs; these models appear to be more robust for meta-analyses of single proportions compared with Freeman-Tukey double arcsine transformation.^3^ Heterogeneity was measured using the *I*^2^ statistic, with thresholds of 25%, 50%, and 75% indicating low, moderate, and high heterogeneity, respectively. All analyses were performed using the metafor package in R software, version 4.0.2 (R Foundation for Statistical Computing). Statistical significance was set at 2-tailed *P* = .05.

## Results

**Figure 1.**
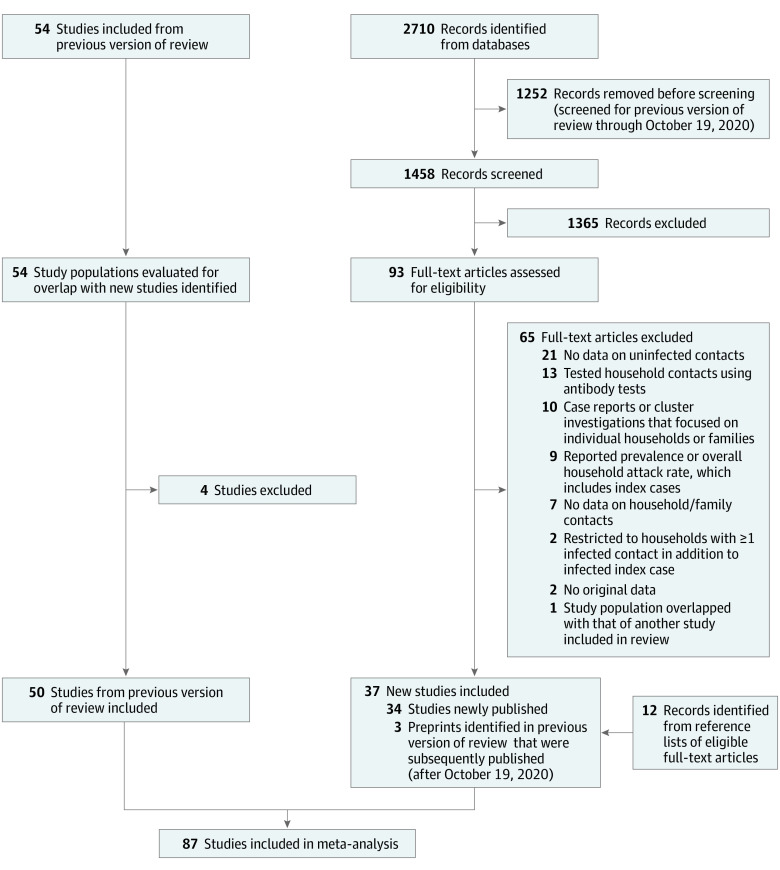
PRISMA Flow Diagram

**Figure 2.**
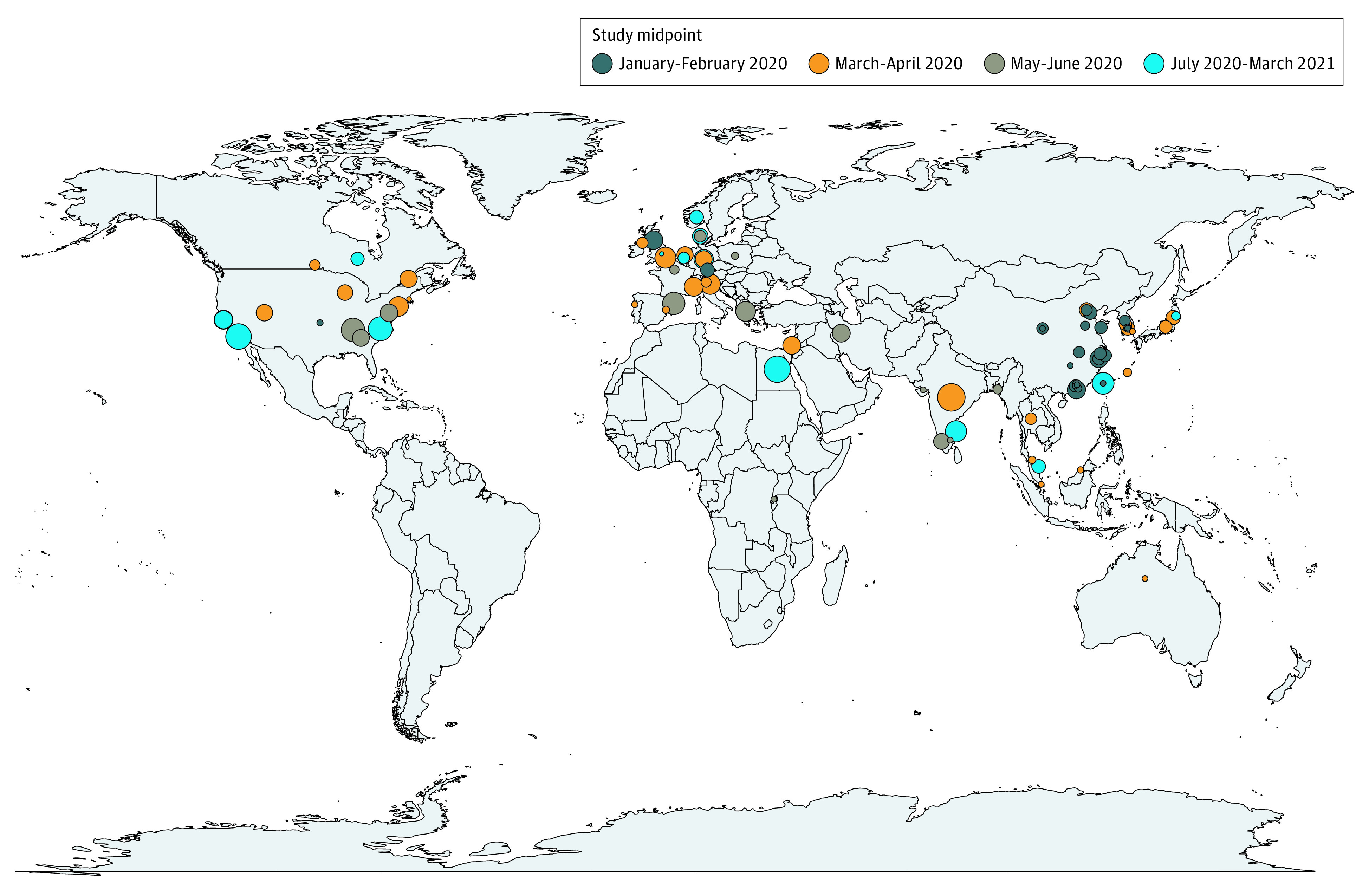
Household Secondary Attack Rates by Study Location For studies that included data from multiple regions within a country, a point in the center of the country was selected. Circle sizes represent extent of secondary attack rates, with small circles indicating 0.2, medium circles indicating 0.4, and large circles indicating 0.6.

We identified 2722 records (2710 records from database searches and 12 records from the reference lists of eligible articles) published between October 20, 2020, and June 17, 2021; of those, 93 full-text articles reporting household secondary transmission of SARS-CoV-2 were assessed for eligibility, and 37 studies^4,5,6,7,8,9,10,11,12,13,14,15,16,17,18,19,20,21,22,23,24,25,26,27,28,29,30,31,32,33,34,35,36,37,38,39,40^ were eligible for inclusion (3 of these studies were preprints that were identified in our previous review and subsequently published) (Figure 1; eTable 1 in the Supplement). These 37 new studies were combined with 50 of the 54 studies (published through October 19, 2020) included in our previous review (4 studies^41,42,43,44^ from Wuhan, China, were excluded because their study populations overlapped with another recent study),^14^ resulting in 87 total studies^4,5,6,7,8,9,10,11,12,13,14,15,16,17,18,19,20,21,22,23,24,25,26,27,28,29,30,31,32,33,34,35,36,37,38,39,40,45,46,47,48,49,50,51,52,53,54,55,56,57,58,59,60,61,62,63,64,65,66,67,68,69,70,71,72,73,74,75,76,77,78,79,80,81,82,83,84,85,86,87,88,89,90,91,92,93,94^ representing 1 249 163 household contacts from 30 countries. The estimated overall household SAR for all 87 studies was 18.9% (95% CI, 16.2%-22.0%), with significant heterogeneity (*I^2^* = 99.4%; *P* < .001) (Figure 2). Excluding studies with only asymptomatic^85^ or pediatric^36,66^ index cases, studies that tested only^7,9,15,17,19,24,26,29,30,31,35,37,45,47,61,65,68,69,71,77,79,81,82,86,87,90,92,94^ or asymptomatic^78^ contacts, studies with long follow-up periods (≥21 days),^5,8,9,23,46,92^ and studies published as preprints,^8,23,24,29,45,79,88,89,90,92^ the overall SAR among the 47 remaining studies^4,6,10,11,12,13,14,16,18,20,21,22,25,27,28,32,33,34,38,39,48,49,50,51,52,53,54,55,57,58,59,60,62,63,64,67,70,72,73,74,75,76,80,83,84,91,93^ was 19.9% (95% CI, 16.2%-24.2%).

**Figure 3.**
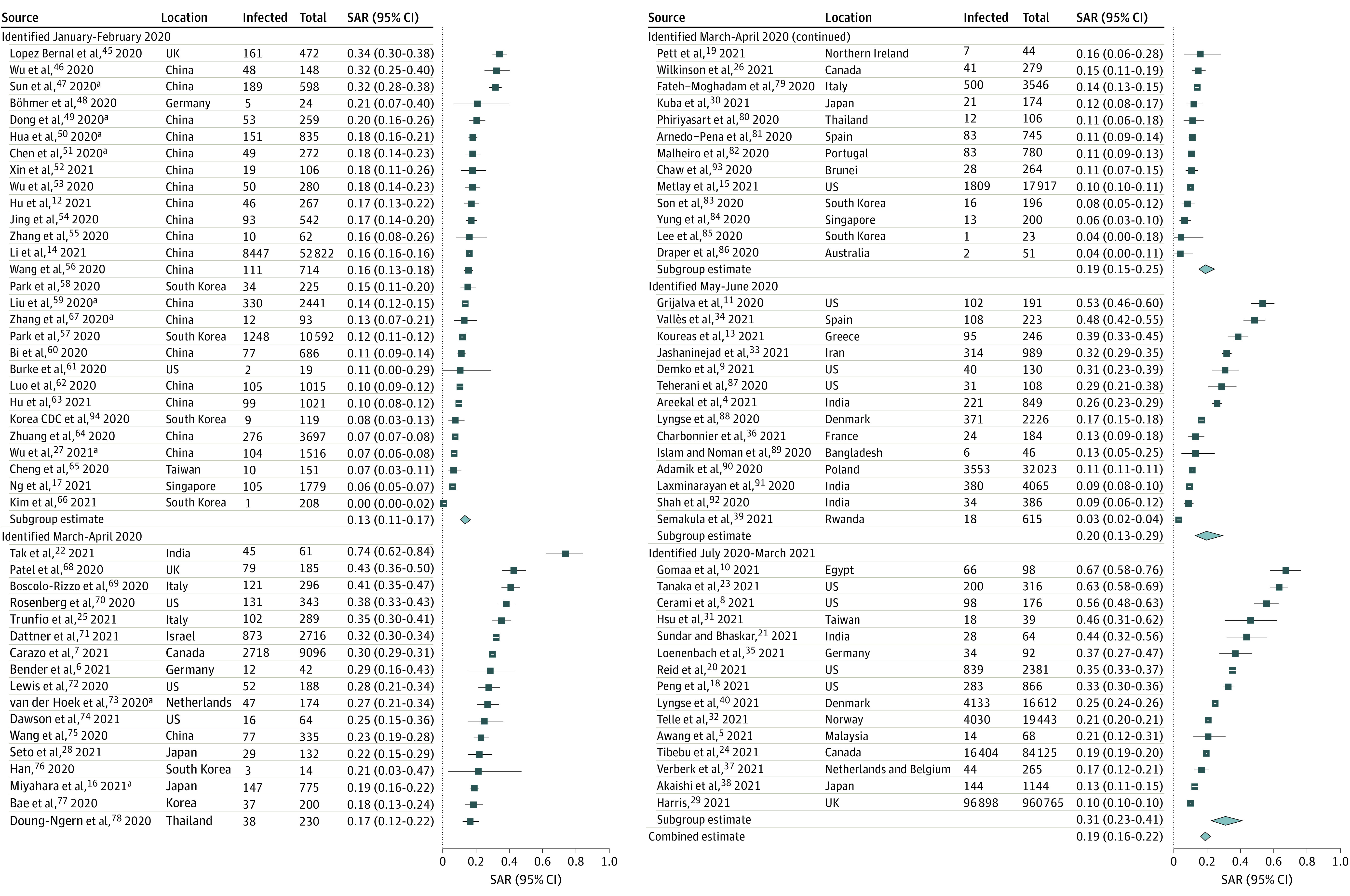
Household Secondary Attack Rates by Midpoint of Index Case Identification Period For studies that spanned multiple months, the midpoint was used. For example, when the index case identification period for all households was December 2019 to April 2020, the midpoint was February 2020, and the study was categorized as January to February 2020. The meta-analysis excluded 4 studies from Wuhan, China,^41,42,43,44^ that had overlapping populations with Li et al.^14^ Point sizes are an inverse function of the precision of the estimates, and bars correspond to 95% CIs. Diamonds represent summary SAR estimates with corresponding 95% CIs. ^a^Study included family contacts, which may have comprised individuals outside the household.

When analyzing household SAR by study period, we observed an increasing pattern over time. Compared with the SAR for 28 studies^12,14,17,27,45,46,47,48,49,50,51,52,53,54,55,56,57,58,59,60,61,62,63,64,65,66,67,94^ from January to February 2020 (13.4%; 95% CI, 10.7%-16.7%), the SAR was significantly higher for 30 studies^6,7,15,16,19,22,25,26,28,30,68,69,70,71,72,73,74,75,76,77,78,79,80,81,82,83,84,85,86,93^ from March to April 2020 (19.4%; 95% CI, 15.2%-24.5%; *P* = .03) and 15 studies^5,8,10,18,20,21,23,24,29,31,32,35,37,38,40^ from July 2020 to March 2021 (31.1%; 95% CI, 22.6%-41.1%; *P* < .001) but not significantly different from the SAR for 14 studies^4,9,11,13,33,34,36,39,87,88,89,90,91,92^ from May to June 2020 (19.9%; 95% CI, 13.0%-29.3%;* P* = .07) (Figure 3^14^). To elucidate factors associated with differences in SAR, we explored attributes of studies from the periods with the lowest and highest household SARs. Among 28 studies^12,14,17,27,45,46,47,48,49,50,51,52,53,54,55,56,57,58,59,60,61,62,63,64,65,66,67,94^ from January to February 2020 and 15 studies^5,8,10,18,20,21,23,24,29,31,32,35,37,38,40^ from July 2020 to March 2021, 6 studies^12,46,54,57,59,62^ (21.4%) and 4 studies^8,10,20,23^ (25.0%), respectively, reported testing contacts at least twice, 1 study^46^ (3.6%) and 3 studies^5,8,23^ (18.8%) reported following contacts for longer than 14 days, 1 study^45^ (3.6%) and 6 studies^8,23,24,29,37,40^ (33.3%) were published as preprints, 21 studies^12,14,27,46,48,49,50,51,52,53,54,55,57,58,59,60,62,63,64,66,67^ (75.0%) and 10 studies^5,8,10,18,20,21,23,32,38,40^ (66.6%) tested all contacts regardless of symptoms, and 0 studies and 3 studies^18,35,40^ (18.8%) reported SARs for variants of concern (VOCs).

The SARs were significantly higher for adult contacts (29.9%; 95% CI, 24.0%-36.6%) than for child contacts (17.5%; 95% CI, 12.6%-23.7%; *P* < .001),^7,8,11,13,14,15,26,30,32,35,40,45,46,50,54,60,70,71,72,73,75,87,88,91^ for spousal contacts (39.8%; 95% CI, 30.0%-50.5%) than for other household contacts (18.3%; 95% CI, 12.1%-26.7%; *P* = .001),^8,11,17,30,33,46,47,52,72,93,95^ for contacts with comorbidities (50.0%; 95% CI, 41.4%-58.6%) than for contacts without comorbidities (22.0%; 95% CI, 13.4%-33.9%; *P* = .04),^30,45,46^ in symptomatic index cases (20.2%; 95% CI, 13.9%-28.3%)^6,13,14,16,24,27,58,93^ than in asymptomatic (3.0%; 95% CI, 1.7%-5.4%)^6,14,24,27,58,93^ or presymptomatic (8.1%; 95% CI, 7.3%-9.1%; *P* < .001)^24,58,93^ index cases, and in households with 1 contact (35.5%; 95% CI, 26.2%-46.2%) than in households with 3 or more contacts (21.2%; 95% CI, 14.8%-29.4%; *P* = .02)^11,16,30,32,40,41,45,46,70,81,88^ (Table). The SARs were not associated with the contact’s sex^8,11,13,14,15,17,26,28,30,33,40,45,46,47,52,54,72,81,84,88,91^ or ethnicity^11,18,72^ or with the index case’s age,^11,13,14,16,24,32,35,57,91^ sex,^11,13,14,16,24,32,46,52,72,81,84,91^ presence of fever,^11,46,52^ or presence of cough.^11,46,52^ When the analysis was restricted to laboratory-confirmed results,^30,45,46^ the estimated SAR to contacts with comorbidities was 43.9% (95% CI, 32.1%-56.5%). The estimated mean SAR for the B.1.1.7 (α) variant was 24.5% (95% CI, 10.9%-46.2%),^35,40,96^ with significant heterogeneity (*I^2^* = 99.5%; *P* < .001) (eFigure in the Supplement). Restricting the analysis to studies with a more uniform design,^11,16,32,70^ SARs were not significantly different for the number of contacts in the household (*P* = .51) (eTable 2 in the Supplement). No studies with data regarding the comorbidity covariate met the criteria for inclusion in this subanalysis.

**Table.** Characteristics of Studies Included in Analysis of Household Secondary Attack Rates for SARS-CoV-2

Characteristic	Studies, No.	SAR, % (95% CI)
Measures used for overall SAR assessment		
Laboratory-confirmed results plus probable untested symptomatic cases	87^4,5,6,7,8,9,10,11,12,13,14,15,16,17,18,19,20,21,22,23,24,25,26,27,28,29,30,31,32,33,34,35,36,37,38,39,40,45,46,47,48,49,50,51,52,53,54,55,56,57,58,59,60,61,62,63,64,65,66,67,68,69,70,71,72,73,74,75,76,77,78,79,80,81,82,83,84,85,86,87,88,89,90,91,92,93,94^^a^	18.9 (16.2-22.0)
Laboratory-confirmed results only	81^4,5,6,8,9,10,11,12,13,14,15,16,17,18,20,21,22,23,25,26,27,28,29,30,31,32,33,34,35,36,37,38,39,40,45,46,47,48,49,50,51,52,53,54,55,56,57,58,59,60,61,62,63,64,65,66,67,69,70,71,72,73,74,75,76,77,78,79,80,82,83,84,85,86,88,89,90,91,92,93,94^^a^	18.1 (15.4-21.3)
Contact age		
Adults (≥18 y)	24^7,8,11,13,14,15,26,30,32,35,40,45,46,50,54,60,70,71,72,73,75,87,88,91^^b^	29.9 (24.0-36.6)
Children (<18 y)	24^7,8,11,13,14,15,26,30,32,35,40,45,46,50,54,60,70,71,72,73,75,87,88,91^^b^	17.5 (12.6-23.7)
Contact sex		
Female	21^8,11,13,14,15,17,26,28,30,33,40,45,46,47,52,54,72,81,84,88,91^^b^	22.4 (17.4-28.5)
Male	21^8,11,13,14,15,17,26,28,30,33,40,45,46,47,52,54,72,81,84,88,91^^b^	20.2 (15.2-26.4)
Contact ethnicity^c^		
Hispanic or Latino	3^11,18,72^	36.0 (16.7-61.2)
Non-Hispanic or non-Latino	3^11,18,72^	36.4 (25.7-48.8)
Contact comorbidities		
Any	3^30,45,46^	50.0 (41.4-58.6)
None indicated	3^30,45,46^	22.0 (13.4-33.9)
Relationship to index case		
Spouse	11^8,11,17,30,33,46,47,52,72,93,95^	39.8 (30.0-50.5)
Other	11^8,11,17,30,33,46,47,52,72,93,95^	18.3 (12.1-26.7)
Index case age		
Adult (≥18 y)	9^11,13,14,16,24,32,35,57,91^	22.7 (15.2-32.6)
Child (<18 y)	9^11,13,14,16,24,32,35,57,91^	18.5 (11.8-27.7)
Index case sex		
Female	12^11,13,14,16,24,32,46,52,72,81,84,91^^b^	22.3 (15.8-30.5)
Male	12^11,13,14,16,24,32,46,52,72,81,84,91^^b^	21.3 (15.1-29.2)
Index case symptom status^d^		
Symptomatic	8^6,13,14,16,24,27,58,93^	20.2 (13.9-28.3)
Asymptomatic	6^6,14,24,27,58,93^	3.0 (1.7-5.4)
Presymptomatic	3^24,58,93^	8.1 (7.3-9.1)
Asymptomatic and/or presymptomatic	8^6,13,14,16,24,27,58,93^	3.9 (2.1-6.8)
Index case fever		
Yes	3^11,46,52^	20.6 (12.2-32.7)
No	3^11,46,52^	14.7 (10.6-19.9)
Index case cough		
Yes	3^11,46,52^	22.7 (11.3-40.3)
No	3^11,46,52^	17.3 (13.9-21.4)
Contacts in household, No.		
1	11^11,16,30,32,40,41,45,46,70,81,88^	35.5 (26.2-46.2)
2	11^11,16,30,32,40,41,45,46,70,81,88^	31.8 (20.4-45.9)
≥3	11^11,16,30,32,40,41,45,46,70,81,88^	21.2 (14.8-29.4)
Location		
China or Singapore	22^12,14,17,27,46,47,49,50,51,52,53,54,55,56,59,60,62,63,64,67,75,84^^a^	14.4 (11.8-17.4)
Other	65^4,5,6,7,8,9,10,11,13,15,16,18,19,20,21,22,23,24,25,26,28,29,30,31,32,33,34,35,36,37,38,39,40,45,48,57,58,61,65,66,68,69,70,71,72,73,74,76,77,78,79,80,81,82,83,85,86,87,88,89,90,91,92,93,94^	20.7 (17.0-24.9)
Testing protocol^e^		
Symptomatic and asymptomatic individuals	57^4,5,6,8,10,11,12,13,14,16,18,20,21,22,23,25,27,28,32,33,34,36,38,39,40,46,48,49,50,51,52,53,54,55,57,58,59,60,62,63,64,66,67,70,72,73,74,75,76,80,83,84,85,88,89,91,93^^a^	19.8 (16.1-24.1)
Symptomatic individuals only	28^7,9,15,17,19,24,26,29,30,31,35,37,45,47,61,65,68,69,71,77,79,81,82,86,87,90,92,94^^a^	17.5 (13.6-22.1)
Index case identification period excluding overlapping dates		
December 2019-April 2020	52^6,12,14,17,19,22,25,26,27,45,46,47,48,49,50,51,52,53,54,55,56,57,58,59,60,61,62,63,64,65,66,67,68,69,70,72,73,74,75,76,77,78,79,80,81,82,83,84,85,86,93,94^^a^	15.8 (13.0-19.1)
July 2020-March 2021	14^4,5,18,20,21,23,24,29,33,34,35,36,38,88^	27.7 (20.6-36.2)
Study published as preprint		
Yes	12^8,23,24,29,37,40,45,79,88,89,90,92^	21.0 (13.8-30.6)
No	75^4,5,6,7,9,10,11,12,13,14,15,16,17,18,19,20,21,22,25,26,27,28,30,31,32,33,34,35,36,37,38,39,46,47,48,49,50,51,52,53,54,55,56,57,58,59,60,61,62,63,64,65,66,67,68,69,70,71,72,74,75,76,77,78,80,81,82,83,84,85,86,87,91,93,94^	18.6 (15.7-21.9)
Restriction to studies testing all contacts at least twice	15^8,10,11,12,20,23,34,39,46,54,57,59,62,73,80^^b^	26.2 (16.5-39.0)
Restriction to studies with long follow-up duration (≥21 d)	6^5,8,9,23,46,92^	32.3 (18.0-51.0)
Proportion of households with any secondary transmission	15^7,8,9,13,17,26,30,37,46,70,72,75,84,86,92^	35.0 (22.8-49.6)

Abbreviation: SAR, secondary attack rate.

^a^ Excludes 4 studies^41,42,43,44^ from Wuhan, China, that had populations overlapping with Li et al.^14^

^b^ Excludes 1 study^44^ from Wuhan, China, that had populations overlapping with Li et al.^14^

^c^ Restricted to studies in the US.

^d^ Restricted to studies that disaggregated SARs for at least 2 of the following: symptomatic, presymptomatic, and asymptomatic individuals.

^e^ Excludes 2 studies,^56,78^ 1 in which the testing protocol could not be determined^56^ and 1 in which only asymptomatic contacts received testing.^78^

## Discussion

This updated systematic review and meta-analysis found that, with the addition of 37 studies,^4,5,6,7,8,9,10,11,12,13,14,15,16,17,18,19,20,21,22,23,24,25,26,27,28,29,30,31,32,33,34,35,36,37,38,39,40^ the estimated overall household SAR of SARS-CoV-2 was 18.9%, which is similar to the estimate in the previous review.^1^ Nonetheless, when analyzing SAR by study period, we observed an increase in household transmission over time. Potential explanations for this temporal pattern include improved diagnostic procedures and tools, longer follow-up (which may have captured tertiary transmission or transmission from nonhousehold contacts), more contagious variants, and different study locations. We found lower SARs in studies from China and Singapore,^17,84,97^ potentially owing to mandated quarantine policies. It is also conceivable that the higher SARs observed may be a reflection of publication and time-trend biases, which can impact the generalizability of living systematic reviews.^98^

Results from the subgroup analyses reported in our previous systematic review and meta-analysis^1^ remained largely similar, with a few exceptions. We observed higher transmission to contacts with comorbidities across 3 studies.^30,45,46^ Two of these studies^30,45^ tested only symptomatic contacts. It is possible that testing was more common among symptomatic contacts with comorbidities.^99^ Individuals with comorbidities may also be more susceptible to SARS-CoV-2 infection via a number of molecular mechanisms.^100^ For example, Metlay et al^15^ reported that SARs were highest to household contacts with liver disease (25.5%), kidney disease (24.0%), and hypertension (21.6%). There was also a higher estimate of transmission from asymptomatic or presymptomatic index cases across 8 total studies^6,13,14,16,24,27,58,93^ compared with the transmission found in the previous meta-analysis,^1^ although this transmission remained considerably lower than transmission from symptomatic index cases. Studies of household transmission frequently combine these groups; however, another systematic review^101^ that included nonhousehold contacts reported higher transmission from presymptomatic index cases (7%; 95% CI, 3%-11%; 11 studies) than from asymptomatic index cases (1%; 95% CI, 0%-2%; 10 studies). Presymptomatic SAR is based on overall exposure before symptom onset, and presymptomatic exposure is usually of substantially shorter duration than symptomatic exposure. Most studies reporting SARs from symptomatic index cases have not separated the different phases of exposure but have combined the presymptomatic and symptomatic phases (eg, Areekal et al,^4^ Sundar and Bhaskar,^21^ and Valles et al^34^). This approach may partially account for lower SARs among presymptomatic index cases. Many studies included in our systematic review cautioned that they may not have identified both asymptomatic index cases and asymptomatic household contacts.

Several recent studies^18,35,40,88,96,102,103,104,105,106^ examined household SAR by viral variant. We limited our meta-analyses of variants to only those that were reported in 3 or more studies, which only included the B.1.1.7 (α) variant. For the B.1.1.7 (α) variant, SARs ranged from 9.0% to 42.0%^35,40,96,102,103^ and were reported to be higher compared with SARs for wild-type variants^102^ or non-VOCs^104^ in Ontario, Canada, and compared with SARs for other lineages in the Netherlands^88^ and Oslo, Norway,^103^ but lower compared with SARs for the B.1.617.2 (δ) variant in England.^96^ These findings are consistent with those reported in a modeling study^105^ that estimated that the transmissibility of the B.1.1.7 (α) variant was 43% to 90% higher than that of preexisting variants.

Regarding variants that were examined in fewer than 3 studies for which we did not perform meta-analyses, SARs were also higher for the B.1.351 (β) or P.1 (γ) variant (27.2%) and non-VOC variants (23.3%) compared with wild-type variants in Ontario, Canada.^102^Household SARs were higher for contacts with the B.1.427 and B.1.429 (ε) variants (35.6%) compared with contacts without these variants in San Francisco, California,^18^ whereas no major differences in household SARs were found between individuals with the B.1.526 (ι) variant and non-VOCs in New York, New York.^106^

Emerging data suggest that vaccination may not only be associated with the prevention of SARS-CoV-2 infections among vaccinated individuals but may also be associated with reductions in transmission to unvaccinated household contacts.^29,107,108^ A recent study^29^ (published as a preprint) of more than 1 million household contacts in England found that, compared with households in which no individuals received COVID-19 vaccines, household SARs were 40% to 50% lower among households in which index cases received BNT162b2 (Pfizer–BioNTech) or ChAdOx1 nCoV-19 (Oxford-AstraZeneca) vaccines 21 days or more before receiving a positive test result for SARS-CoV-2. Another study^108^ (published as a preprint) of almost 200 000 household members in Scotland reported a 30% reduction in COVID-19 cases among household contacts of health care workers who received BNT162b2 or ChAdOx1 nCoV-19 vaccines at 14 days or more after the second dose compared with household contacts of health care workers who did not receive these vaccines. These findings are consistent with those of a study conducted in Finland^107^ that suggested indirect benefit of 8.7% (95% CI, −28.9% to 35.4%) at 2 weeks and 42.9% (95% CI, 22.3%-58.1%) at 10 weeks after the first dose of BNT162b2 or mRNA-1273 vaccines. Results suggesting a possible association between vaccination and reductions in infectiousness include lower disease severity, shorter duration of symptoms, and lower viral load.^109^

### Limitations

This study has limitations. As described in the previous systematic review and meta-analysis,^1^ there was high heterogeneity across studies, which may be attributable to differences in study design (eg, follow-up duration, frequency of testing, and universal and/or symptomatic testing), transmission mitigation strategies after index case diagnosis, household crowding, underlying seroprevalence, and other factors. There was insufficient information to perform meta-analyses of SARs by other VOCs.

## Conclusions

This updated systematic review and meta-analysis suggests that the household remains an important site of SARS-CoV-2 transmission, and recent studies have reported higher household SAR estimates compared with the earliest reports. More transmissible variants may be associated with further changes. Recent data suggest that 1 dose of a COVID-19 vaccine may be associated with reductions in the risk of household transmission by up to 50%,^29^ potentially supporting the case for universal vaccination and offering a path forward to protect household contacts.

## Notes

Supplement. **eTable 1.** Description of Studies Published From October 20, 2020, to June 17, 2021 **eTable 2.** Household Secondary Attack Rates for SARS-CoV-2, Restricted to Studies With a More Uniform Design **eFigure.** Household Secondary Attack Rates of SARS-CoV-2 for B.1.1.7 (α) Variant
eReferences
