# Supplementary material for: Factors Associated With Household Transmission of SARS-CoV-2: An Updated Systematic Review and Meta-analysis
Source: JAMA Netw Open. 2021 Aug 27;4(8):e2122240. doi: 10.1001/jamanetworkopen.2021.22240 (PMC8397928; doi:10.1001/jamanetworkopen.2021.22240)
Supplement: Supplement. — eTable 1. Description of Studies Published From October 20, 2020, to June 17, 2021 eTable 2. Household Secondary Attack Rates for SARS-CoV-2, Restricted to Studies With a More Uniform Design eFigure. Household Secondary Attack Rates of SARS-CoV-2 for B.1.1.7 (α) Variant eReferences [file jamanetwopen-e2122240-s001.pdf]

Supplementary Online Content

Madewell ZJ, Yang Y, Longini IM Jr, Halloran ME, Dean NE. Factors associated with household transmission of SARS-CoV-2: an updated systematic review and meta-analysis. *JAMA Netw Open*. 2021;4(8):e2122240. doi:10.1001/jamanetworkopen.2021.22240

eTable 1. Description of Studies Published From October 20, 2020, to June 17, 2021

eTable 2. Household Secondary Attack Rates for SARS-CoV-2, Restricted to Studies With a More Uniform Design

eFigure. Household Secondary Attack Rates of SARS-CoV-2 for B.1.1.7 (α) Variant

eReferences

This supplementary material has been provided by the authors to give readers additional information about their work.

© 2021 Madewell ZJ et al. *JAMA Network Open*.

eTable 1. Description of Studies Published From October 20, 2020, to June 17, 2021

Authors	Journal	Location	Study period	No. index cases	Index case symptom status	Contact type (household, family)	Duration of follow-up (days)	Test used to diagnose contacts	Universal testing or only symptomatic	Number of tests per contact	Overall SAR (infected/total)
Akaishi et al.[1]	Scientific Reports	Miyagi Prefecture, Japan	July, 2020 – March, 2021	–	Symptomatic and asymptomatic	Household	14	RT-PCR	Universal	1	12.6% (144/1,144)
Areekal et al.[2]	Journal of Clinical & Diagnostic Research	Kerala, India	June–July, 2020	267	Symptomatic and asymptomatic	Household	14	RT-PCR	Universal	1	26.0% (221/849)
Awang et al.[3]	Infectious Diseases	Terengganu, Malaysia	October 22–November 15, 2020	–	Symptomatic	Household	21	RT-PCR	Universal	1	20.6% (14/68)
Bender et al.[4]	Emerging Infectious Diseases	Southern Germany	March, 2020	–	Symptomatic and asymptomatic	Household	14	Laboratory test or symptom-based	Universal	1	Lab test only: 12.5% (4/32)<br><br>Respiratory symptoms: 28.6% (12/42)
Carazo et al.[5]	Infection Control & Hospital Epidemiology	Quebec, Canada	March 1–June 14, 2020	3823	Symptomatic	Household	–	Symptom-based; No testing	Symptom-based	–	29.8% (2,718/9,096)
Cerami et al.[6]	Preprint	North Carolina, USA	April 29–October 16, 2020	99	Symptomatic	Household	28	RT-PCR and antibody test	Universal	4 PCR	PCR + antibody test: 60.2% (106/176)

© 2021 Madewell ZJ et al. *JAMA Network Open*.

											PCR only: 73 positive at baseline + 25 more during follow-up=98 55.7% (98/176)
Charbonnier et al.[7]	Frontiers in Pediatrics	Paris, France	May 8–July 27, 2020	34	Symptomatic and asymptomatic	Household	14	RT-PCR	Universal	1	PCR only: 13.0% (24/184)
Demko et al.[8]	Open Forum Infectious Diseases	Baltimore, USA	April 21–July 23, 2020	74	72/74 were symptomatic	Household	28	RT-PCR	Symptomatic	1	53.4% (102/191)
Gomaa et al.[9]	PLOS Pathogens	Egypt	April–October, 2020	23	Symptomatic	Household	14	RT-PCR and antibody test	Universal	5 PCR	PCR + antibody test: 89.8% (88/98)<br><br>PCR only: 67.3% (66/98)
Grijalva et al.[10]	Morbidity and Mortality Weekly Report	Tennessee & Wisconsin, USA	April–September, 2020	101	Symptomatic	Household	14	RT-PCR	Universal	Multiple	53.4% (102/191)
Harris et al.[11]	Preprint	England	January 4–February 28, 2021	365,447-4,107-20,110=341,230	Symptomatic	Household	14	RT-PCR	Symptomatic	–	Unvaccinated index cases: 10.1% (96,898/960,765)
Hsu et al.[12]	Journal of the Formosan	Taiwan	January 28, 2020 to –	26	–	Household	14	RT-PCR	Symptomatic	1	46.2% (18/39)

© 2021 Madewell ZJ et al. *JAMA Network Open*.

	Medical Association		February 28, 2021								
Hu et al.[13]	International Journal of Infectious Diseases	Guangzhou, China	January–March, 2020	100	Symptomatic	Household	14	RT-PCR	Universal	At least twice	17.2% (46/267)
Jashaninejad et al.[14]	Journal of Research in Health Sciences	Hamadan, Iran	Mid-May–mid-July, 2020	323	Symptomatic and asymptomatic	Household	14	RT-PCR	Universal	1	31.7% (314/989)
Koureas et al.[15]	International Journal of Environmental Research and Public Health	Thessaly, Greece	April 8–June 4, 2020	135	Symptomatic and asymptomatic	Household	–	RT-PCR	Universal	–	38.6% (95/246)
Kuba et al.[16]	Japanese Journal of Infectious Diseases	Okinawa, Japan	February 14–May 31, 2020	78	Symptomatic	Household	14	RT-PCR	Symptomatic	1	12.1% (21/174)
Li et al.[17]	The Lancet Infectious Diseases	Wuhan, China	December 2, 2019–April 18, 2020	29578	Symptomatic and asymptomatic	Household	14	RT-PCR	Universal	1	16.0% (8,447/52,822)
Loenenbach et al.[18]	Eurosurveillance	Germany	January–February, 2021	38	Symptomatic and asymptomatic	Household	At least 7	RT-PCR	Symptomatic	1	37.0% (34/92)
Lyngse et al.[19]	Preprint	Denmark	January 11–February 7, 2021	8,093	–	Household	14	RT-PCR and whole genome sequencing	–	–	24.9% (4,133/16,612)

© 2021 Madewell ZJ et al. *JAMA Network Open*.

Metlay et al.[20]	JAMA Network Open	Boston, USA	March–May, 2020	7262	Symptomatic	Household	–	–	Symptomatic	–	10.1% (1,809/17,917)
Miyahara et al.[21]	Emerging Infectious Diseases	Japan	February 22–May 31, 2020	306	Symptomatic and asymptomatic	Family	14	RT-PCR	Universal	1	19.0% (147/775)
Ng et al.[22]	The Lancet Infectious Diseases	Singapore	January 23–April 3, 2020	581	Symptomatic	Household	14	RT-PCR	Symptomatic	1	5.9% (105/1,779)
Peng et al.[23]	Clinical Infectious Diseases	San Francisco, USA	January, 2021	319	Symptomatic and asymptomatic	Household	–	Genome sequencing	Universal	1	32.3% (283/867)
Pett et al.[24]	Epidemiology & Infection	Northern Ireland	February 26–April 26, 2020	27	Symptomatic	Household	14	–	Symptomatic	Symptom based	15.9% (7/44)
Reid et al.[25]	Open Forum Infectious Diseases	San Francisco, USA	June 8–August 31, 2020	–	Symptomatic	Household	14	RT-PCR	Universal	2	35.2% (839/2,381)
Semakula et al.[26]	BMJ Global Health	Rwanda	March 14 –July 20, 2020	–	Symptomatic and asymptomatic	Household	7–14	RT-PCR	Universal	At least twice	2.9% (18/615)
Seto et al.[27]	Japanese Journal of Infectious Diseases	Yamagata, Japan	January 3–May 31, 2020	69	Symptomatic and asymptomatic	Household	14	RT-PCR	Universal	1	22.0% (29/132)
Sundar et al.[28]	Germs	Chennai, India	August 1–20, 2020	18	Symptomatic	Household	–	RT-PCR	Universal	1	43.7% (28/64)
Tak et al.[29]	Infectious Disease Modelling	India	March 25–April 24, 2020	–	Symptomatic	Household	–	RT-PCR	Universal	1	73.8% (45/61)
Tanaka et al.[30]	Preprint	Los Angeles, USA	June 17–Decemb	135	Symptomatic or	Household	28	RT-PCR and	Universal	6	PCR + antibody tests:

© 2021 Madewell ZJ et al. *JAMA Network Open*.

			er 31, 2020		asymptomatic			antibody test			77.7% reported in Results<br><br>238 secondary cases + 78 non-cases=316 total contacts reported in Table 2. PCR only SAR = 63.2% (reported in Results). Therefore, 316*.632=200; SAR=63.2% (200/316)
Telle et al.[31]	European Journal of Epidemiology	Norway	March 1, 2020–January 31, 2021	7548	Symptomatic and asymptomatic	Household	7	RT-PCR	Universal	1	SAR7=20.7% (4,030/19,443)
Tibebu et al.[32]	Preprint	Ontario, Canada	July 1–November 30, 2020	29352	Symptomatic and asymptomatic	Household	14-28	Symptom-based; No testing	Symptom based	–	19.5% (16,404/84,125)
Trunfio et al.[33]	Frontiers	Turin, Italy	March, 2020	132	Symptomatic and asymptomatic	Household	–	RT-PCR or symptom-based	Universal	1	PCR only (176 tested): 25.6% (74/289)

© 2021 Madewell ZJ et al. *JAMA Network Open*.

											PCR + "signs and symptoms suggestive of COVID-19" after the onset of the linked index case= 35.3% (102/289)
Vallès et al.[34]	Public Health	Barcelona, Spain	May–July 2020,	81	Symptomatic	Household	14	RT-PCR	Universal	2	223 household members * .483=108 48.3% (108/223)
Verberk et al.[35]	Preprint	Netherlands and Belgium	April 20–December 2, 2020	117	Symptomatic and asymptomatic	Household	21	RT-PCR and antibody test	Symptomatic	1	PCR + antibody test: 27.9% (74/265)<br><br>PCR only: 16.6% (44/265)
Wilkinson et al.[36]	Canadian Journal of Public Health	Winnipeg, Canada	mid-March–April 28, 2020	102	Symptomatic	Household	14	–	Symptomatic	–	14.7% (41/279)
Wu et al.[37]	Clinical Infectious Diseases	China	January 5–April 7, 2020	578	Symptomatic and asymptomatic	Household	14	RT-PCR	Universal	1	6.9% (104/1,516)

© 2021 Madewell ZJ et al. *JAMA Network Open*.

eTable 2. Household Secondary Attack Rates for SARS-CoV-2, Restricted to Studies With a More Uniform Design[a]

	No. studies	SAR (95% CI)
Overall SAR		
Laboratory confirmed + probable untested symptomatic cases	47[b]	19.9% (16.2–24.2%)
Laboratory confirmed results only	47[b]	19.4% (15.8–23.6%)
Contact age		
Adults (≥18 years)	12[c]	28.3% (19.6–39.0%)
Children (<18 years)	12[c]	17.9% (11.6–26.5%)
Contact sex		
Female	10[c]	23.0% (16.4–31.2%)
Male	10[c]	19.3% (12.5–28.6%)
Contact ethnicity[d]		
Hispanic/Latino	3	36.0% (16.7–61.2%)
Non-Hispanic/Latino	3	36.4% (25.7–48.8%)
Relationship to index case		
Spouse	6	32.5% (24.9–41.3%)
Other	6	16.2% (10.6–24.1%)
Index case age		
Adults	7	22.2% (13.4–34.5%)
Children	7	16.5% (9.7–26.6%)
Index case sex		
Female	9[c]	23.5% (15.3–34.2%)
Male	9[c]	21.8% (14.3–31.7%)
Index case symptom status[e]		
Symptomatic	7	19.9% (12.9–29.4%)
Asymptomatic	5	2.0% (1.5–2.8%)
Presymptomatic	2	5.2% (2.0–13.0%)
Asymptomatic/presymptomatic	7	3.3% (1.7–6.4%)
Index case fever		
Yes	2	26.4% (11.5–22.9%)
No	2	13.2% (9.2–18.5%)
Index case cough		
Yes	2	15.3% (10.5–21.8%)
No	2	17.3% (13.6–21.7%)
Number of contacts in household		
1	4	32.6% (15.4–56.4%)
2	4	36.6% (18.7–59.1%)

© 2021 Madewell ZJ et al. *JAMA Network Open*.

≥3	4	28.7% (19.6–39.9%)
Location		
China or Singapore	18[b]	13.6% (11.4–16.1%)
Other	29	24.9% (19.0–31.9%)
Index case identification time period excluding overlapping dates		
December, 2019 – April, 2020	32[b]	16.9% (13.7–20.7%)
July, 2020 – March, 2021	7	31.3% (23.1–40.9%)
Restricted to studies that tested all contacts at least twice	12[c]	21.5% (12.6–34.1%)
Proportion of households with any secondary transmission	6	37.8% (20.0–59.6%)

SAR: secondary attack rate; CI: confidence interval

[a] Excludes studies with only asymptomatic[38] or pediatric[7,39] index cases, that tested only symptomatic[5,8,11,12,16,18,20,22,24,32,35,36,40-55] or asymptomatic[56] contacts, with long follow-up (≥21 days),[3,6,8,30,52,57] and preprints.[6,11,30,32,40,48,49,52,58,59]

[b] Excludes four studies from original analysis from Wuhan[60-63] that had overlapping populations with Li *et al*.[17]

[c] Excludes one study from original analysis from Wuhan[63] that had overlapping populations with Li *et al*.[17]

[d] Restricted to studies in the United States.

[e] Restricted to studies that disaggregated secondary attack rates for at least two of: symptomatic, presymptomatic, and asymptomatic.

© 2021 Madewell ZJ et al. *JAMA Network Open*.

eFigure. Household Secondary Attack Rates of SARS-CoV-2 for B.1.1.7 (α) Variant

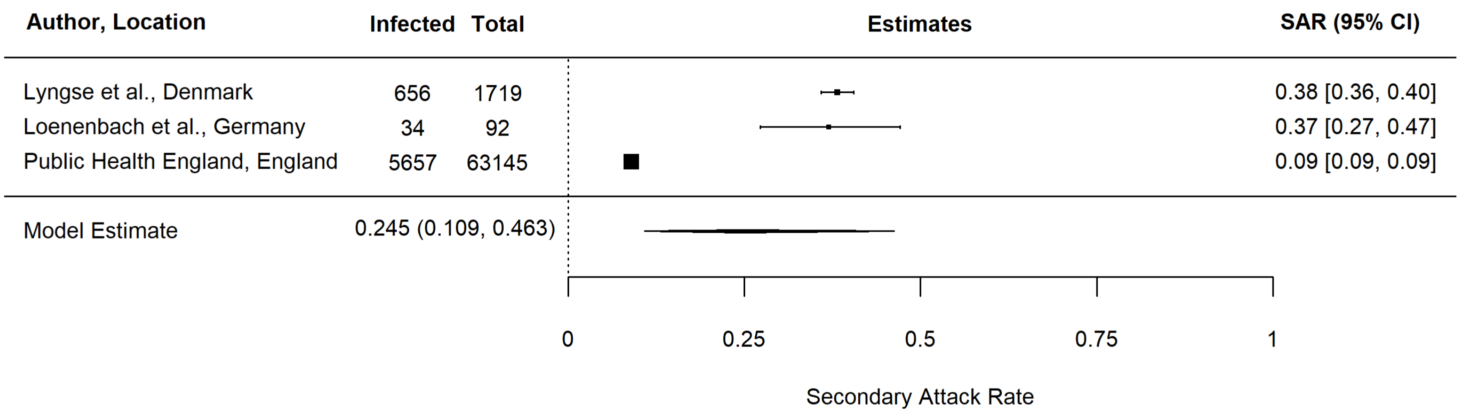

Point sizes are an inverse function of the precision of the estimates and bars correspond to 95% confidence intervals.

© 2021 Madewell ZJ et al. *JAMA Network Open*.

eReferences

1. Akaishi T, Kushimoto S, Katori Y, et al. COVID-19 transmission in group living environments and households. *Scientific Reports.* 2021;11(1):11616.
2. Areekal B, Vijayan S, Suseela MS, et al. Risk Factors, Epidemiological and Clinical Outcome of Close Contacts of COVID-19 Cases in a Tertiary Hospital in Southern India. *Journal of Clinical & Diagnostic Research.* 2021;15(3).
3. Awang H, Yaacob EL, Syed Aluawi SN, et al. A case–control study of determinants for COVID-19 infection based on contact tracing in Dungun district, Terengganu state of Malaysia. *Infectious Diseases.* 2021;53(3):222-225.
4. Bender JK, Brandl M, Höhle M, Buchholz U, Zeitlmann N. Analysis of asymptomatic and presymptomatic transmission in SARS-CoV-2 outbreak, Germany, 2020. *Emerging infectious diseases.* 2021;27(4):1159.
5. Carazo S, Laliberté D, Villeneuve J, et al. Characterization and evolution of infection control practices among SARS-CoV-2 infected healthcare workers of acute care hospitals and long-term care facilities in Quebec, Canada, Spring 2020. *Infection Control & Hospital Epidemiology.* 2021:1-37.
6. Cerami C, Rapp T, Lin F-C, et al. High household transmission of SARS-CoV-2 in the United States: living density, viral load, and disproportionate impact on communities of color. *medRxiv.* 2021:2021.2003.2010.21253173.
7. Charbonnier L, Rouprêt-Serzec J, Caseris M, et al. Contribution of Serological Rapid Diagnostic Tests to the Strategy of Contact Tracing in Households Following SARS-CoV-2 Infection Diagnosis in Children. *Frontiers in Pediatrics.* 2021;9(217).
8. Demko ZO, Antar AAR, Blair PW, et al. Clustering of SARS-CoV-2 infections in households of patients diagnosed in the outpatient setting in Baltimore, MD. *Open Forum Infectious Diseases.* 2021.
9. Gomaa MR, El Rifay AS, Shehata M, et al. Incidence, household transmission, and neutralizing antibody seroprevalence of Coronavirus Disease 2019 in Egypt: Results of a community-based cohort. *PLOS Pathogens.* 2021;17(3):e1009413.
10. Grijalva CG, Rolfes MA, Zhu Y, et al. Transmission of SARS-COV-2 infections in households—Tennessee and Wisconsin, April–September 2020. *Morbidity and Mortality Weekly Report.* 2020;69(44):1631.
11. Harris RJ, Hall JA, Zaidi A, Andrews NJ, Dunbar JK, Dabrera G. Impact of vaccination on household transmission of SARS-COV-2 in England *[preprint].* 2021.
12. Hsu C-Y, Wang J-T, Huang K-C, Chiao-Hsin Fan A, Yeh Y-P, Li-Sheng Chen S. Household Transmission but without the Community-acquired Outbreak of COVID-19 in Taiwan. *Journal of the Formosan Medical Association.* 2021.
13. Hu P, Ma M, Jing Q, et al. Retrospective study identifies infection related risk factors in close contacts during COVID-19 epidemic. *International Journal of Infectious Diseases.* 2021;103:395-401.
14. Jashaninejad R, Doosti-Irani A, Karami M, Keramat F, Mirzaei M. Transmission of COVID-19 and its Determinants among Close Contacts of COVID-19 Patients Running title. *Journal of Research in Health Sciences.* 2021.
15. Koureas M, Speletas M, Bogogiannidou Z, et al. Transmission Dynamics of SARS-CoV-2 during an Outbreak in a Roma Community in Thessaly, Greece—Control Measures and Lessons Learned. *International Journal of Environmental Research and Public Health.* 2021;18(6):2878.
16. Kuba Y, Shingaki A, Nidaira M, et al. The characteristics of household transmission during COVID-19 outbreak in Okinawa, Japan from February to May 2020. *Japanese journal of infectious diseases.* 2021:JJID. 2020.2943.

© 2021 Madewell ZJ et al. *JAMA Network Open*.

17. Li F, Li Y-Y, Liu M-J, et al. Household transmission of SARS-CoV-2 and risk factors for susceptibility and infectivity in Wuhan: a retrospective observational study. *The Lancet Infectious Diseases.* 2021.
18. Loenenbach A, Markus I, Lehfeld A-S, et al. SARS-CoV-2 variant B.1.1.7 susceptibility and infectiousness of children and adults deduced from investigations of childcare centre outbreaks, Germany, 2021. *Eurosurveillance.* 2021;26(21):2100433.
19. Lyngse FP, Mølbak K, Skov RL, et al. Increased Transmissibility of SARS-CoV-2 Lineage B.1.1.7 by Age and Viral Load: Evidence from Danish Households. *medRxiv.* 2021:2021.2004.2016.21255459.
20. Metlay JP, Haas JS, Soltoff AE, Armstrong KA. Household Transmission of SARS-CoV-2. *JAMA Network Open.* 2021;4(2):e210304-e210304.
21. Miyahara R, Tsuchiya N, Yasuda I, et al. Familial Clusters of Coronavirus Disease in 10 Prefectures, Japan, February− May 2020. *Emerging infectious diseases.* 2021;27(3):915.
22. Ng OT, Marimuthu K, Koh V, et al. SARS-CoV-2 seroprevalence and transmission risk factors among high-risk close contacts: a retrospective cohort study. *The Lancet Infectious Diseases.* 2021;21(3):333-343.
23. Peng J, Liu J, Mann SA, et al. Estimation of secondary household attack rates for emergent spike L452R SARS-CoV-2 variants detected by genomic surveillance at a community-based testing site in San Francisco. *Clinical infectious diseases : an official publication of the Infectious Diseases Society of America.* 2021.
24. Pett J, McAleavey P, McGurnaghan P, et al. Epidemiology of COVID-19 in Northern Ireland, 26 February 2020–26 April 2020. *Epidemiology & Infection.* 2021;149.
25. Reid MJA, Prado P, Brosnan H, et al. Assessing testing strategies and duration of quarantine in contact tracing for SARS-CoV-2: a retrospective study of San Francisco's COVID-19 contact tracing program, June- August, 2020. *Open Forum Infectious Diseases.* 2021.
26. Semakula M, Niragire F, Umutoni A, et al. The secondary transmission pattern of COVID-19 based on contact tracing in Rwanda. *BMJ Global Health.* 2021;6(6):e004885.
27. Seto J, Aoki Y, Komabayashi K, et al. Epidemiology of coronavirus disease 2019 in Yamagata Prefecture, Japan, January-May 2020: The importance of retrospective contact tracing. *Japanese journal of infectious diseases.* 2021.
28. Sunday V, Bhaskar E. Low secondary transmission rates of SARS-CoV-2 infection among contacts of construction laborers at open air environment. *Germs.* 2021;11(1):128-131.
29. Tak P, Rohilla J. COVID-19 contact tracing in a tertiary care hospital: A retrospective chart review. *Infectious Disease Modelling.* 2021;6:1-4.
30. Tanaka ML, Marentes Ruiz CJ, Malhotra S, et al. Urban Household Transmission of SARS-CoV-2 During Periods of High and Low Community Transmission.
31. Telle K, Jorgensen SB, Hart RK, Greve-Isdahl M, Kacelnik O. Secondary attack rates of COVID-19 in Norwegian families: a nation-wide register-based study. *European Journal of Epidemiology.* 2021.
32. Tibebu S, A. Brown K, Daneman N, Paul LA, Buchan SA. Household secondary attack rate of COVID-19 by household size and index case characteristics. *medRxiv.* 2021:2021.2002.2023.21252287.
33. Trunfio M, Longo BM, Alladio F, et al. On the SARS-CoV-2 "Variolation Hypothesis": No Association Between Viral Load of Index Cases and COVID-19 Severity of Secondary Cases. *Frontiers in Microbiology.* 2021;12(473).
34. Vallès X, Roure S, Valerio L, et al. SARS-CoV-2 contact tracing among disadvantaged populations during epidemic intervals should be a priority strategy: results from a pilot experiment in Barcelona. *Public Health.* 2021.

© 2021 Madewell ZJ et al. *JAMA Network Open*.

35. Verberk J, de Hoog M, Westerhof I, et al. Transmission of SARS-CoV-2 within households: a prospective cohort study in the Netherlands and Belgium – Interim results. *medRxiv.* 2021:2021.2004.2023.21255846.
36. Wilkinson K, Chen X, Shaw S. Secondary attack rate of COVID-19 in household contacts in the Winnipeg Health Region, Canada. *Canadian Journal of Public Health.* 2021;112(1):12-16.
37. Wu P, Liu F, Chang Z, et al. Assessing asymptomatic, pre-symptomatic and symptomatic transmission risk of SARS-CoV-2. *Clinical Infectious Diseases.* 2021.
38. Lee M, Eun Y, Park K, Heo J, Son H. Follow up investigation of asymptomatic COVID-19 cases at diagnosis in Busan, Korea. *Epidemiology and Health.* 2020;0(0):e2020046-2020040.
39. Kim J, Choe YJ, Lee J, et al. Role of children in household transmission of COVID-19. *Archives of Disease in Childhood.* 2020:archdischild-2020-319910.
40. Adamik B, Bawiec M, Bezborodov V, et al. Bounds on the total number of SARS-CoV-2 infections: The link between severeness rate, household attack rate and the number of undetected cases. 2020.
41. Arnedo-Pena A, Sabater-Vidal S, Meseguer-Ferrer N, et al. COVID-19 secondary attack rate and risk factors in household contacts in Castellon (Spain): Preliminary report. *Enfermedades Emergentes.* 2020;19(2):64-70.
42. Bae S, Kim H, Jung T-Y, et al. Epidemiological Characteristics of COVID-19 Outbreak at Fitness Centers in Cheonan, Korea. *J Korean Med Sci.* 2020;35(31).
43. Boscolo-Rizzo P, Borsetto D, Spinato G, et al. New onset of loss of smell or taste in household contacts of home-isolated SARS-CoV-2-positive subjects. *European Archives of Oto-rhino-laryngology.* 2020:1-4.
44. Burke RM. Active monitoring of persons exposed to patients with confirmed COVID-19—United States, January–February 2020. *MMWR Morbidity and mortality weekly report.* 2020;69.
45. Cheng HY, Jian SW, Liu DP, Ng TC, Huang WT, Lin HH. Contact Tracing Assessment of COVID-19 Transmission Dynamics in Taiwan and Risk at Different Exposure Periods Before and After Symptom Onset. *JAMA Internal Medicine.* 2020.
46. Dattner I, Goldberg Y, Katriel G, et al. The role of children in the spread of COVID-19: Using household data from Bnei Brak, Israel, to estimate the relative susceptibility and infectivity of children. *PLoS computational biology.* 2021;17(2):e1008559.
47. Draper AD, Dempsey KE, Boyd RH, et al. The first 2 months of COVID-19 contact tracing in the Northern Territory of Australia, March-April 2020. *Communicable Diseases Intelligence.* 2020;44.
48. Fateh-Moghadam P, Battisti L, Molinaro S, et al. Contact tracing during Phase I of the COVID-19 pandemic in the Province of Trento, Italy: key findings and recommendations. *medRxiv.* 2020.
49. Lopez Bernal J, Panagiotopoulos N, Byers C, et al. Transmission dynamics of COVID-19 in household and community settings in the United Kingdom. *medRxiv.* 2020:2020.2008.2019.20177188.
50. Malheiro R, Figueiredo AL, Magalhães JP, et al. Effectiveness of contact tracing and quarantine on reducing COVID-19 transmission: a retrospective cohort study. *Public Health.* 2020.
51. Patel A, Charani E, Ariyanayagam D, et al. New-onset anosmia and ageusia in adult patients diagnosed with SARS-CoV-2 infection. *Clinical Microbiology and Infection.* 2020.
52. Shah K, Desai N, Saxena D, Mavalankar D, Mishra U, Patel GC. Household Secondary Attack Rate in Gandhinagar district of Gujarat state from Western India. *medRxiv.* 2020:2020.2009.2003.20187336.

© 2021 Madewell ZJ et al. *JAMA Network Open*.

53. Sun WW, Ling F, Pan JR, et al. [Epidemiological characteristics of 2019 novel coronavirus family clustering in Zhejiang Province]. *Zhonghua Yu Fang Yi Xue Za Zhi.* 2020;54(0):E027.
54. Teherani MF, Kao CM, Camacho-Gonzalez A, et al. Burden of illness in households with SARS-CoV-2 infected children. *Journal of the Pediatric Infectious Diseases Society.* 2020.
55. Covid-19 National Emergency Response Center Epidemiology Case Management Team Korea Centers for Disease Control Prevention. Coronavirus Disease-19: Summary of 2,370 Contact Investigations of the First 30 Cases in the Republic of Korea. *Osong Public Health and Research Perspectives.* 2020;11(2):81-84.
56. Doung-ngern P, Suphanchaimat R, Panjagampatthana A, et al. Case-Control Study of Use of Personal Protective Measures and Risk for Severe Acute Respiratory Syndrome Coronavirus 2 Infection, Thailand. *Emerging Infectious Diseases.* 2020;26(11).
57. Wu J, Huang Y, Tu C, et al. Household Transmission of SARS-CoV-2, Zhuhai, China, 2020. *Clinical Infectious Diseases.* 2020.
58. Islam SS, Noman ASM. Transmission Dynamics and Contact Tracing Assessment of COVID-19 in Chattogram, Bangladesh and Potential Risk of Close Contacts at Different Exposure Settings. *Bangladesh and Potential Risk of Close Contacts at Different Exposure Settings.*
59. Lyngse FP, Kirkeby CT, Halasa T, et al. COVID-19 Transmission Within Danish Households: A Nationwide Study from Lockdown to Reopening. *medRxiv.* 2020.
60. Wang Z, Ma W, Zheng X, Wu G, Zhang R. Household transmission of SARS-CoV-2. *The Journal of Infection.* 2020.
61. Wang X, Zhou Q, He Y, et al. Nosocomial outbreak of COVID-19 pneumonia in Wuhan, China. *The European Respiratory Journal.* 2020;55(6).
62. Yu HJ, Hu YF, Liu XX, et al. Household infection: The predominant risk factor for close contacts of patients with COVID-19. *Travel Medicine and Infectious Disease.* 2020;36:101809.
63. Li W, Zhang B, Lu J, et al. Characteristics of household transmission of COVID-19. *Clinical Infectious Diseases.* 2020;71(8):1943-1946.

© 2021 Madewell ZJ et al. *JAMA Network Open*.
